# Basal bodies in *Xenopus*

**DOI:** 10.1186/s13630-016-0024-6

**Published:** 2016-02-03

**Authors:** Siwei Zhang, Brian J. Mitchell

**Affiliations:** Department of Cell and Molecular Biology, Feinberg School of Medicine, Northwestern University, Chicago, IL 60611 USA

**Keywords:** *Xenopus*, Basal body, Centriole, Deuterosome, Ciliogenesis, Cilia, Multiciliated cells

## Abstract

*Xenopus* has been one of the earliest and most important vertebrate model organisms for investigating the role and structure of basal bodies. Early transmission electron microscopy studies in *Xenopus* revealed the fine structures of *Xenopus* basal bodies and their accessory structures. Subsequent investigations using multiciliated cells in the *Xenopus* epidermis have further revealed many important features regarding the transcriptional regulation of basal body amplification as well as the regulation of basal body/cilia polarity. Future basal body research using *Xenopus* is expected to focus on the application of modern genome editing techniques (CRISPR/TALEN) to characterize the components of basal body proteins and their molecular functions.

## The organism

The term *Xenopus* refers to a collection of approximately 20 fully aquatic frog species within the genus *Xenopus*. In scientific classifications, they belong to Kingdom Animalia, Phylum Chordata, Class Amphibia, Order Anura, and Family Pipidae. The early *Xenopus* embryo, due to their large size and free development outside the mother’s body, have been one of the most important models for the investigation of early vertebrate development as well as basic biology for many years [1]. Initial investigations using the *Xenopus* species, which can be dated back to the mid-late 1800s, are mainly restricted to the larger, easy-to-handle *X. laevis* strain. However, a genome duplication event during the evolution of *X. laevis* has been discovered, which indicates that *X. laevis* is a pseudotetraploid species with genetic redundancy [2]. This has prevented detailed genetic studies to be performed on the *Xenopus* species. However, a diploid *Xenopus* system *X. (Silurana) tropicalis* with a much smaller genome size was introduced allowing detailed genetic manipulations to be performed in *Xenopus* [3, 4]. Recently, the full genome sequence of both *X. laevis* and *X. tropicalis* has been released to provide the basis for advanced genomic manipulations, such as CRISPR/Cas9 and/or TALEN, in addition to the traditional morpholino oligo (MO) knockdown approach [5].

## Basic basal body structure

**Fig. 1:**
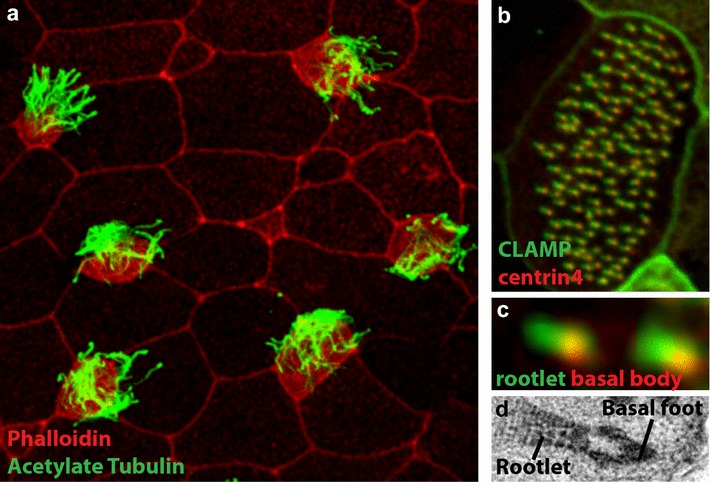
Multiciliated epithelium of Xenopus. **a** The punctate pattern of MCCs in the skin of Xenopus embryos with cilia marked with anti-acetylated tubulin (*green*) and cell boundaries marked with phalloidin (*red*). **b** Single MCC with the basal bodies marked with Centrin4-RFP (*red*) and the rootlets marked with GFP-CLAMP (*green*). **c** Close-up image of two basal body/rootlet pairs from (**b**). **d** TEM image showing the rootlet and the basal foot in opposite orientation surrounding the basal body. In all images, anterior is to the *left* and posterior is to the *right*. The effective stroke of the ciliary beat is oriented to the posterior

The basal body is a specialized form of centriole that is located at the cell membrane and provides the anchoring point as well as the growth basis for the cilium. As with most of the organisms that possess centrioles during their cell cycle, the main structure of the *Xenopus* basal body is the characteristic 9 blades of microtubule triplets [6, 7]. *Xenopus* has proven to be an extremely powerful system for the analysis of cilia formation and function [6]. Unfortunately, relative to many other model organisms, there is a paucity of transmission electron microscopy (TEM)-level detail of structural information, particularly for the basal body. Despite the fact that most internal organs possess primary cilium, our literature search found no structural information regarding the basal bodies of these cilia. Likewise, the gastrocoel roof plate (GRP) in *Xenopus* is an analogous structure to the mouse embryonic node (or Kupffer’s vesicle in zebrafish), and it possesses motile mono-cilia which generate the directional flow that establishes embryonic left–right patterning [7]. Again, no structural detail of these basal bodies and cilia is currently available. However, it is reasonable to assume that, similar to what has been found in other organisms, the structure of the ciliary axoneme of immotile and motile mono-cilia differs significantly. Another special case that is worth mentioning is the outer segments (OS) of both rod and cone photoreceptors found in *Xenopus* retina, which arise as an elaboration of an immotile primary cilia. In mature *Xenopus* retina, this modified ciliary structure remains as the sole cytoplasmic connection that bridges the inner segment and OS of the photoreceptors [8]. Unfortunately, no ultrastructural detail of this ciliary axoneme is available. Perhaps the most well-studied cilia in *Xenopus* are the motile cilia present in the multiciliated cells (MCCs) that occur on the surface of the early embryo (Fig. 1). Each MCC possesses approximately 150 basal bodies that will nucleate their motile cilia. For the purposes of this primer, we will restrict our discussion to this particular form of basal body.

The basal bodies of *Xenopus* MCCs can be labeled by the strong presence of γ-tubulin during both live imaging and immunostaining [9]. Several additional tubulin genes, in addition to the α-, β-, and γ-tubulin genes found in most organisms, have been identified in the *Xenopus* system. The δ-tubulin gene (*tubd1, tubulin, delta 1*) gene has been identified in *X. laevis* by the Stearns lab [10]. The ε-tubulin (*tube1, tubulin, epsilon 1*) gene has also been identified in *X. laevis* for its roles in centriole duplication and microtubule organization [11, 12]. In addition, a special tubulin gene, ζ-tubulin (*tubz1, tubulin, zeta 1*), has also been characterized as an important component of the basal foot in MCCs. In contrast, in cycling cells, ζ-tubulin does not locate to centrioles but rather associates with the TRiC/CCT cytoplasmic chaperone complex in the cytoplasm [13]. Interestingly, from an evolutionary point of view, the latter three tubulin families form a co-conserved module, named the ZED module. This ZED module has been independently lost in several branches of the evolution tree, such as in higher fungi, higher plants, and placenta mammals. It is also important to note that for the species that possess the ZED module, ε-tubulin gene is always present, while there is a chance of losing either δ- or ζ-tubulin, but not both [13]. It has been proposed that the presence of the ZED module may be essential for the formation of centriolar appendages; however, further investigations will be required to resolve this question.

## Additional basal body structures or accessory structures

**Fig. 2:**
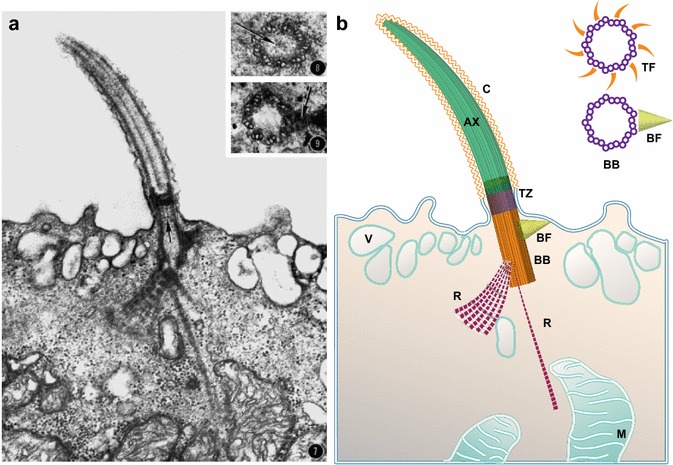
Vertical cross section of a Xenopus motile cilium. **a** TEM image of a single cilium with drawing representation of individual structures **b** *AX* axoneme, *BB* basal body, *BF* basal foot, *C* cilium, *M* mitochondria, *R* rootlet, *TZ* transition zone, *TF* transition fibers, *V* vesicles. In the *figure*, anterior is to the *left* and posterior is to the *right*. *Image* in **a** was taken with permission from Steinmann 1968

The systematic study of *Xenopus* basal bodies and their accessory structures first started in the late 1960s [14]. Similar to other vertebrate species, there are transition fibers, whose structure is similar to distal appendages, that help anchor the basal body to the membrane in the transition zone (Fig. 2, inlay) [15]. Most cells that possess an immotile primary cilium are characterized by the association of a daughter centriole positioned orthogonal to the basal body throughout the lifetime of the cilia [16]. In contrast, in MCCs, the basal bodies of motile cilia are free-standing structures with no associated daughter centrioles [17, 18]. During maturation, centrioles acquire appendages such as transitional fibers and basal feet that serve as anchoring structures to stabilize the basal bodies [19, 20]. In mature, polarized MCCs, the basal foot projects posteriorly orthogonal to the basal body in the direction of the ciliary effective stroke (Fig. 2, inlay). Both ζ- and ε-tubulin have been shown to localize at the basal foot in *Xenopus*, and the foot serves as a microtubule organizing center (MTOC) [13, 21–23]. In contrast, another accessory structure, the striated rootlet, projects anteriorly away from the basal body and is always positioned 180 ° opposite from the basal foot (Fig. 1). The rootlet is a large striated, fibrous structure that extends from the basal body into the cytoplasm and provides structural support to the cilium, as well as demarcating the orientation of motile cilium. This rootlet is prominent in many TEM images of MCC basal bodies and can be visualized using light microscopy based on the localization of rootlet associated proteins such as Mig12 and Spef1/CLAMP (Figs. 1b–d, 2) [24, 25]. The structure of the rootlet is complex, sometimes containing multiple branches (Fig. 2) [14–26]. While the rootlet is generally thought to serve as a mechanical anchor for the beating cilium, it is possible that it also provides a scaffold for signaling events. The Wnt/PCP signaling molecule Dvl2 localizes adjacent to the centriole along the rootlet [24]. Similarly, several focal adhesion proteins including FAK, Vinculin, and Paxillin associate with the proximal and distal ends of the rootlet and are likely to provide certain tension based signaling [27]. Finally, while the basal foot associates primarily with microtubules, the rootlet appears to interact with the dense sub-apical actin network surrounding these cilia [21].

## The origins of basal body

The basal bodies in *Xenopus* are both converted from centrioles as well as built *de novo* depending on the cell type. For cells that generate a single cilium, such as cells found in the GRP and gut, basal bodies are converted from the mother centriole similar to other systems [28]. While cycling cells contain both a mother and a daughter centriole, typically it is only the older “mother” centriole that has gone through a full cell cycle that is competent to become a basal body. Interestingly, ectopic over-expression of Foxj1, a protein that plays a crucial role during the differentiation and maintenance of ciliated cells, is able to drive basal body conversion inducing the formation of 1–2 cilia per cell when expressed in non-ciliated epithelial cells [29]. This phenomenon suggests that, in the *Xenopus* skin, both the mother and daughter centrioles may maintain a certain level of basal body competency. In MCCs that generate dozens of basal bodies, the process appears to be quite distinct. Instead of nucleating from an older “mother” centriole, the vast majority of basal bodies nucleate from a structure termed the deuterosome [30]. The regulation of this process is still poorly understood but clearly requires the key centriole duplication regulating proteins Plk4 and Cep152 [30, 31]. Remarkably, while these cells are post-mitotic, the nascent centrioles are immediately competent to become cilia-nucleating basal bodies without going through a cell cycle. As soon as centrioles are generated, they begin their migration to the apical cell surface and immediately initiate cilia formation. How this centriole-to-basal body conversion is regulated remains a mystery.

While the structures of centrioles and basal bodies are generally comparable, there are important structural and functional distinctions between them. For example, microtubules (part of the ciliary axoneme) directly and specifically emerge from the distal end of the basal body, whereas cytoplasmic and mitotic microtubules nucleate in all directions from the pericentriolar material surrounding the centriolar pair of the centrosome. In addition to the basal body-specific appendages detailed above, another important distinction between centrioles and basal bodies is their relationships with cell membranes. Basal bodies associate with membrane-bound vesicles as the vesicles migrate to and fuse with the apical cell membrane [24]. This membrane association is critical to basal body function and components of the basal body-linked transition zone and is thought to regulate distinct membrane compartments. More detailed reviews on this topic, including the structural and functional differences between ciliary membrane and cell membrane, are provided in [32, 33].

## The life cycle of basal body and its other functions

**Fig. 3:**
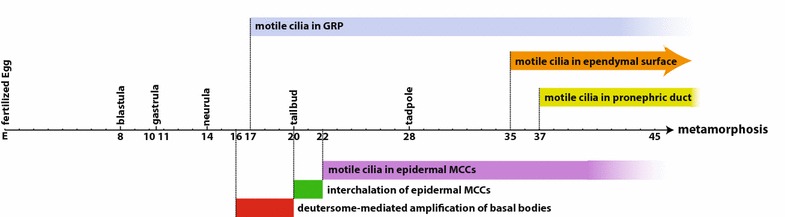
The time axis showing the first appearance of the various types of basal bodies/cilia at different developmental stages through Xenopus early development

Most cells in *Xenopus* contain a centriolar pair, or centrosome that functions as the MTOC and is critical during mitosis. *Xenopus* do not possess basal bodies through all stages of their life cycle. During early development when cells are rapidly dividing they do not possess primary cilium during interphase. In fact, cells with primary cilia have not been widely described and have only been reported in the GRP, the neural tube, and later during organogenesis (Fig. 3) [7, 29, 34–39]. Most of the early embryonic cells do not have cilia, and the formation of basal bodies occurs at distinct times in different tissues (Fig. 3). The massive centriole amplification which occurs in MCCs begins around stages 16–17, and these cells typically complete ciliogenesis between stage 20 and 22. Prior to becoming basal bodies and forming cilia, the numerous centrioles of MCCs are essential for nucleating a pool of acetylated stable microtubules that is important for the process of radial intercalation, by which MCCs insert into the outer epithelium [40].

## Identification of basal body components

To date, no studies that systematically address the protein components of *Xenopus* basal bodies have been identified. However, numerous proteins are known to localize to basal bodies, including many proteins that localize to centrioles in other systems, and the components seem quite comparable to other vertebrate (and non-vertebrate) centrioles. In addition, gene expression analyses in MCCs indicate the up-regulation of many centriolar components that are most certainly contained in the basal bodies of motile cilia [29, 34, 41–43]. While many centriolar components (e.g., Centrin, Poc1, HYLS, Sas6, Plk4, Cep152, and numerous others) appear similar to all centrioles, other components (e.g., Dvl2) likely represent unique features of multiciliated basal bodies.

## Notable basal body findings

The ciliated epithelium of *Xenopus* has proven to be a particularly powerful system for the study of cilia and basal bodies (reviewed in [6, 44]). Specifically, this system has provided the first evidence of PCP signaling and fluid flow affecting cilia/basal body polarity [24, 25, 45], the first evidence of a septin-based cilia diffusion barrier [46], the characterization of MCC-specific transcriptional regulators [29, 41–43, 47], the first characterization of miRNA-mediated regulation of basal body duplication and ciliogenesis [48, 49], and the first molecular characterization of the basal body-generating structure the deuterosome [30]. These and many other important discoveries were facilitated by the molecular, embryological, and imaging techniques that are available in *Xenopus* coupled with the fact that the ciliated epithelia develop on the external surface of the embryos rather than inside the organism. Notably, the discoveries in *Xenopus* have been validated in other vertebrate systems [50–53]. In addition, many human genetic defects have been authenticated and more thoroughly characterized using the tools available in *Xenopus* [54–56].

## Strengths and future of basal body research in *Xenopus*

It is a very exciting time to be using *Xenopus* as a model system to study basal bodies. Recent advances in the detailed quantification of both protein and RNA levels across early *Xenopus* development stages promises to facilitate the analysis of many developmental processes including basal body formation and functions [57]. In addition, recent advances in genome editing technologies including TALENs and CRISPR/Cas hold great potential to allow rapid analysis of genetic mutations [54]. Specifically, the ability to couple CRISPR/Cas with homologous recombination to insert either fluorescent markers or specific mutations will greatly enhance our ability to model human disease in *Xenopus.* Important questions that remain to be answered are as follows: how are centriole amplification and centriole-to-basal body conversion regulated in MCCs that are no longer progressing through the cell cycle; what is the driving force of apical migration/insertion of basal bodies; how do basal bodies and their accessory structures interact with the cytoskeleton as well as with the cell cortex; and what are the similarities/differences between the basal bodies of motile and primary cilia. With these new tools in hand, the next few years will certainly lead to many new advances in our understanding of basal body formation and functions.

## Abbreviations

TEM: transmission electron microscopy
MCC: multiciliated cell
MO: morpholino oligo
GRP: gastrocoel roof plate
OS: outer segments
MTOC: microtubule organizing center
PCP: planar cell polarity
